# MUNet: a novel framework for accurate brain tumor segmentation combining UNet and mamba networks

**DOI:** 10.3389/fncom.2025.1513059

**Published:** 2025-01-29

**Authors:** Lijuan Yang, Qiumei Dong, Da Lin, Chunfang Tian, Xinliang Lü

**Affiliations:** ^1^Department of Rheumatology, Inner Mongolia Autonomous Region Hospital of Traditional Chinese Medicine, Hohhot, China; ^2^College of Traditional Chinese Medicine, Inner Mongolia Medical University, Hohhot, China; ^3^School of Mathematical Sciences, Inner Mongolia University, Hohhot, China; ^4^Department of Oncology, Inner Mongolia Autonomous Region Hospital of Traditional Chinese Medicine, Hohhot, China

**Keywords:** brain tumor segmentation, deep learning, MUNet, SD-SSM module, medical image analysis

## Abstract

Brain tumors are one of the major health threats to humans, and their complex pathological features and anatomical structures make accurate segmentation and detection crucial. However, existing models based on Transformers and Convolutional Neural Networks (CNNs) still have limitations in medical image processing. While Transformers are proficient in capturing global features, they suffer from high computational complexity and require large amounts of data for training. On the other hand, CNNs perform well in extracting local features but have limited performance when handling global information. To address these issues, this paper proposes a novel network framework, MUNet, which combines the advantages of UNet and Mamba, specifically designed for brain tumor segmentation. MUNet introduces the SD-SSM module, which effectively captures both global and local features of the image through selective scanning and state-space modeling, significantly improving segmentation accuracy. Additionally, we design the SD-Conv structure, which reduces feature redundancy without increasing model parameters, further enhancing computational efficiency. Finally, we propose a new loss function that combines mIoU loss, Dice loss, and Boundary loss, which improves segmentation overlap, similarity, and boundary accuracy from multiple perspectives. Experimental results show that, on the BraTS2020 dataset, MUNet achieves DSC values of 0.835, 0.915, and 0.823 for enhancing tumor (ET), whole tumor (WT), and tumor core (TC), respectively, and Hausdorff95 scores of 2.421, 3.755, and 6.437. On the BraTS2018 dataset, MUNet achieves DSC values of 0.815, 0.901, and 0.815, with Hausdorff95 scores of 4.389, 6.243, and 6.152, all outperforming existing methods and achieving significant performance improvements. Furthermore, when validated on the independent LGG dataset, MUNet demonstrated excellent generalization ability, proving its effectiveness in various medical imaging scenarios. The code is available at https://github.com/Dalin1977331/MUNet.

## 1 Introduction

Brain tumors are a type of malignant or benign tumor originating from brain cells or metastasizing from other parts of the body, posing a significant threat to human health (Michael et al., 2021; Rezaei, 2021). They exhibit diverse pathological manifestations and progress rapidly, often leading to severe neurological dysfunctions that affect the quality of life and even endanger the lives of patients (Abhisheka et al., 2023; Ibrahim et al., 2020). Due to the heterogeneity, deep location, and complex anatomical structure of brain tumors, early and accurate diagnosis and treatment are crucial for improving prognosis and therapeutic efficacy.

With the widespread application of deep learning in medical image analysis, researchers have leveraged its powerful feature extraction capabilities to achieve automatic segmentation and detection of brain tumors. Deep learning models can utilize large amounts of MRI data to learn the complex features of tumors, enabling accurate and efficient analysis and identification (Zebari et al., 2020; Chaudhury et al., 2022). Notably, structures like UNet have achieved remarkable results in segmentation tasks, making automated detection and segmentation of brain tumors possible. Compared to traditional manual feature extraction methods, these deep learning-based approaches can more precisely capture the shape and boundary characteristics of tumors, providing a robust tool for clinical assistance (Soulami et al., 2021; Houssein et al., 2021).

In recent years, deep learning models based on Transformers and Convolutional Neural Networks (CNNs) have made significant advancements in the field of image segmentation. Transformer architectures, with their self-attention mechanisms, excel at capturing global features in images (Wang et al., 2023). Complementarily, CNNs have unique advantages in extracting local features, enabling the capture of spatial details within images. However, these methods also have certain limitations. CNN models face challenges in processing global information and may easily overlook long-range spatial correlations (Zhu et al., 2024). On the other hand, while Transformers are proficient at capturing global features, their high computational complexity and demand for large-scale training data limit their application in medical image segmentation (Amgad et al., 2022).

As an emerging deep learning structure, the Mamba network has achieved significant success in the field of computer vision. With its efficient feature extraction capabilities and modular design, Mamba has demonstrated strong performance advantages in tasks such as image segmentation, object detection, and image classification (Zhang et al., 2024). Compared to traditional CNN models, the Mamba network excels in multi-scale feature fusion and contextual information capture, allowing it to better adapt to the complexity and diversity of visual data. Moreover, the Mamba structure significantly reduces computational complexity relative to Transformers (Badiezadeh et al., 2024). However, medical images often contain complex textures and structures, especially MRI brain tumor images, which exhibit considerable heterogeneity and irregularity in their internal features. The variation in shape, size, location, and contrast of tumors relative to surrounding normal tissues makes feature extraction highly complex, posing challenges for the Mamba network in accurately segmenting and identifying tumor boundaries (Tang et al., 2024).

In this paper, we propose a novel network framework named MUNet, which combines the advantages of Unet and Mamba, specifically designed for brain tumor segmentation. To achieve this, we design a new SD-SSM block structure that leverages selective scanning and state space modeling to capture both global and local features of the image. Moreover, without increasing the number of parameters, we introduce the SD-Conv structure, which consists of SCConv (Spatial and Channel Reconstruction Convolution) and Depthwise Separable Convolution, aiming to reduce feature redundancy and improve model efficiency. In MUNet, we apply skip connections to the SD-SSM block, fusing the features of the encoder and decoder to retain multi-scale information. Additionally, we design a new loss function that combines mIoU, Dice, and Boundary losses to optimize the overlap, similarity, and boundary accuracy of the segmentation.

Key contributions of this paper include:

- This paper proposes an innovative framework, MUNet, which combines Unet and Mamba, specifically for brain tumor segmentation. By fully integrating the advantages of both, MUNet achieves more precise and efficient segmentation of tumor regions.
- This paper introduces a new SSM-based structure called the SD-SSM Block. Utilizing selective scanning and dual-channel feature extraction, it effectively captures multi-scale global and local features of the image, enhancing segmentation performance.
- This paper presents the SD-Conv structure, which combines SCConv and DW Conv to compress redundant information between features without increasing the number of model parameters, thereby improving the efficiency of feature extraction.
- For the task of brain tumor segmentation, this paper designs a novel loss function that combines mIoU loss, Dice loss, and Boundary loss. This approach optimizes the segmentation's overlap, similarity, and boundary accuracy from multiple perspectives, thereby enhancing the performance of MUNet in brain tumor segmentation.

The remaining structure of this paper is as follows: Section 2 presents the related work, introducing previous works on brain tumor segmentation using Unet and Mamba. Section 3 covers the methodology, providing a detailed explanation of the MUNet model concept. Section 4 describes the experiments, including comparative experiments and ablation studies. Finally, the conclusion summarizes the entire paper.

## 2 Related works

### 2.1 U-Net network and its innovative evolution

In recent years, the U-Net network has made significant progress in the field of medical image analysis. As a fully convolutional neural network (FCN) (Ho et al., 2021), U-Net employs its encoder-decoder symmetric structure to efficiently extract and fuse local and global features from images, showing exceptional performance in image segmentation tasks (Futrega et al., 2021).

To further enhance the performance of U-Net in medical image segmentation, TransUNet (Chen et al., 2021) combines U-Net with the Vision Transformer (ViT) to propose a hybrid segmentation model based on Transformers. TransUNet builds upon the U-Net encoder and utilizes the self-attention mechanism of Transformers to model global features in images. The ViT module (Li et al., 2023b) introduces patch-level feature capturing during the encoding process, leveraging the global attention mechanism to capture long-range dependencies, thereby enhancing the model's understanding of global context in images. SwinUNet (Cao et al., 2022) further integrates U-Net with the Swin Transformer, proposing a more efficient segmentation framework. Swin Transformer is a hierarchical vision transformer model that introduces a sliding window attention mechanism, effectively capturing long-range dependencies in images while reducing computational complexity. By embedding Swin Transformer modules into the encoder and decoder parts of U-Net, SwinUNet enhances the model's multi-scale feature extraction capabilities and global context modeling abilities. This model demonstrates excellent performance in medical image segmentation tasks, particularly for images with rich texture details and complex structures (Walsh et al., 2022).

To further explore the potential of U-Net, the U-Mamba (Lee and Kim, 2024) structure was developed. U-Mamba combines U-Net with the Mamba network, leveraging Mamba's strengths in feature extraction and multi-scale information fusion to improve segmentation accuracy. With its modular design, the Mamba (Xu et al., 2024) network can be flexibly integrated into U-Net's encoder and decoder, enabling the comprehensive extraction and fusion of features at different scales. Moreover, the Mamba network exhibits strong generalization capabilities when handling complex textures and structures, allowing U-Mamba to achieve more precise segmentation results in medical imaging. Similarly, Mamba-Unet integrates U-Net with Vision Mamba (VMamba) (Zhu et al., 2024), fully combining U-Net's context information capturing abilities with the feature expression advantages of VMamba, thus proposing an efficient network suitable for medical image segmentation (Patro and Agneeswaran, 2024).

In this paper, we propose a novel framework that integrates the Mamba structure with UNet. Unlike existing approaches, we introduce the SD-SSM Block, which captures multi-scale features through selective scanning and dual-channel feature extraction. This design enables the model to better balance global feature modeling with detail preservation. Furthermore, we present the SD-Conv structure, which effectively reduces feature redundancy without increasing the number of parameters. This enhancement improves the efficiency of feature representation, enabling the model to achieve superior accuracy and performance in brain tumor segmentation.

### 2.2 Application of deep learning models in brain tumor segmentation

In recent years, brain tumor segmentation models have made significant progress in the field of deep learning (Magadza and Viriri, 2021). ResUNet, for instance, is a model that combines the residual network (ResNet) (Maji et al., 2022) with the U-Net structure. By introducing residual modules, it effectively addresses the gradient vanishing problem in deep networks, significantly enhancing model stability and convergence speed, thus improving the segmentation capability for complex brain tumor features. However, while this residual structure increases the model's expressive capacity, it also introduces higher computational costs, requiring more memory and longer training times. Models based on DenseNet (Belaid et al., 2024) achieve efficient feature transmission through dense connections, fully leveraging multi-level features to improve segmentation accuracy, especially excelling in capturing tumor edge details. Nonetheless, the computational complexity and memory requirements brought by dense connections limit their application in resource-constrained environments. Attention Gated Networks (Chinnam et al., 2022) introduce an attention mechanism, utilizing attention gates to focus on important features related to target regions, significantly improving focus on target areas and enhancing segmentation accuracy in complex backgrounds for brain tumors. However, the incorporation of the attention mechanism also increases the network's complexity, leading to longer inference times. SegNet (Almotairi et al., 2020), as an encoder-decoder structured model, relies on max-pooling indices for upsampling, maintaining high computational efficiency and exhibiting good real-time performance in brain tumor segmentation, making it suitable for scenarios with high requirements for inference speed. However, compared to other models based on advanced convolutional structures, SegNet shows slightly lower accuracy in segmenting complex tumor morphologies. Residual Attention Networks combine residual connections and attention mechanisms to enhance the model's ability to express target region features while capturing both global and local information, and addressing gradient issues in deep network training (Ranjbarzadeh et al., 2021). However, due to the introduction of residual and attention modules, this model has high computational resource requirements during inference and can exhibit certain inference delays (Jyothi and Singh, 2023). In addition, an interpretable model based on U-Net and DenseNet has been designed for the segmentation and classification of brain tumors. This model enhances interpretability and transparency by generating heat maps that highlight the contribution of each region of the input image to the classification output (Wijethilake et al., 2021). This approach not only improves the model's interpretability but also increases its trustworthiness in clinical diagnosis. Although these techniques provide new insights for tumor survival prediction, they also face several challenges. For example, existing models still suffer from poor interpretability and limited generalization ability, which restrict their widespread application in clinical practice. By combining imaging data with genomic information, more dimensions of data can be leveraged to provide a more reliable survival analysis of tumors, further enhancing the accuracy of predictions (Dasanayaka et al., 2022b).

In this paper, we specifically designed MUNet for the task of brain tumor segmentation, overcoming the limitations of traditional CNN and Transformer architectures. MUNet effectively captures both local and global features of tumors, enhancing feature representation while also reducing computational complexity.

## 3 Methods

### 3.1 Preliminaries

State Space Models (SSM) (Zhu et al., 2024) are a framework for modeling sequential data and are capable of capturing long-range dependencies. SSM is widely used in visual tasks for efficiently processing image sequences. It maps input sequences into a hidden state space and models sequences recursively. This section will introduce the SSM modeling process from three aspects: state updates, output generation, and efficient computation.

**State space representation** The basic form of an SSM uses the state vector *h*(*t*) ∈ ℝ^*N*^ to represent the hidden state, mapping an input sequence *x*(*t*) ∈ ℝ to an output sequence *y*(*t*) ∈ ℝ. The state update equation and output equation are as follows:


(1) $$\begin{array}{l} h^{\prime }\left(t\right)=Ah\left(t\right)+Bx\left(t\right), \end{array}$$

(2) $$\begin{array}{l} y\left(t\right)=Ch\left(t\right), \end{array}$$

where: *A* ∈ ℝ^*N*×*N*^ is the state transition matrix; *B* ∈ ℝ^*N*×1^ is the input mapping matrix; *C* ∈ ℝ^1 × *N*^ is the output mapping matrix.

**Discretization and time scale** SSM is often a discretized version of a continuous system, introducing a time-scale parameter Δ to convert a continuous-time state space into a discrete-time state space. To achieve this transformation, a Zero Order Hold (ZOH) is introduced:


(3) $$\bar{A}=\exp(\Delta A),$$

(4) $$\begin{array}{l} \bar{B}={\left(\Delta A\right)}^{-1}\left(\exp\left(\Delta A\right)-I\right)\cdot \Delta B, \end{array}$$

where Ā and $\bar{B}$ are the discretized state transition matrix and input mapping matrix, respectively. The state update equation in the discrete form is:


(5) $$h_{t}=\bar{A}h_{t-1}+\bar{B}x_{t},$$

(6) $$\begin{array}{l} y_{t}=Ch_{t}. \end{array}$$

**Convolutional form and efficient computation** In SSM, the state update process can be converted into a convolutional kernel form through convolution operations. Assuming the input sequence has a length of *M*, the convolution kernel *K* is represented as:


(7) $$K=[CB,C\bar{A}B,\ldots ,C{\bar{A}}^{M-1}B].$$

The output sequence of the SSM can then be computed using the convolution operation as follows:


(8) $$\begin{array}{l} y=x*K, \end{array}$$

where * denotes the convolution operation.

**2D selective scan** The traditional SSM are primarily designed for one-dimensional sequential data, which limits their ability to effectively capture the spatial information inherent in visual tasks. To overcome this challenge, a two-dimensional selective scanning (SS2D) method is introduced to model the 2D features in visual data effectively.

SS2D first divides the input image into a series of patches and arranges them in four directions: left to right, right to left, top to bottom, and bottom to top, generating four independent feature sequences. Let the original feature be *z* and the direction index be *i*. Each directional feature sequence can be represented as:


(9) $$\begin{array}{l} z_{i}=\text{expand}\left(z,i\right), \end{array}$$

where *z*_*i*_ is the feature sequence in the *i*-th direction, and the function expand represents the operation of arranging image patches according to the direction *i*.

In this way, SS2D achieves a global receptive field without significantly increasing the computational complexity, enabling the model to capture the global context of the image. Each generated feature sequence *z*_*i*_ is then processed through the selective scanning state space model, which performs feature extraction and modeling to obtain the processed feature sequence ${\bar{z}}_{i}$:


(10) $$\begin{array}{l} {\bar{z}}_{i}=S6\left(z_{i}\right), \end{array}$$

where *S*6 denotes the selective scanning state space model's operation on the feature sequence.

After processing all the directional feature sequences, SS2D merges the sequences ${\bar{z}}_{1},{\bar{z}}_{2},{\bar{z}}_{3},{\bar{z}}_{4}$ to reconstruct the 2D feature representation:


(11) $$\begin{array}{l} \bar{z}=\text{merge}\left({\bar{z}}_{1},{\bar{z}}_{2},{\bar{z}}_{3},{\bar{z}}_{4}\right), \end{array}$$

where the function merge denotes the fusion of the features from the four directions to form the final 2D feature representation.

By scanning from four different directions, processing the feature sequences, and merging them, SS2D effectively captures the global spatial information of the image, thereby enhancing the model's perception and understanding of visual tasks. The resulting feature $\bar{z}$ contains rich contextual relationships, providing a comprehensive and efficient representation for subsequent visual analysis tasks.

### 3.2 Model structure

**Figure 1:**
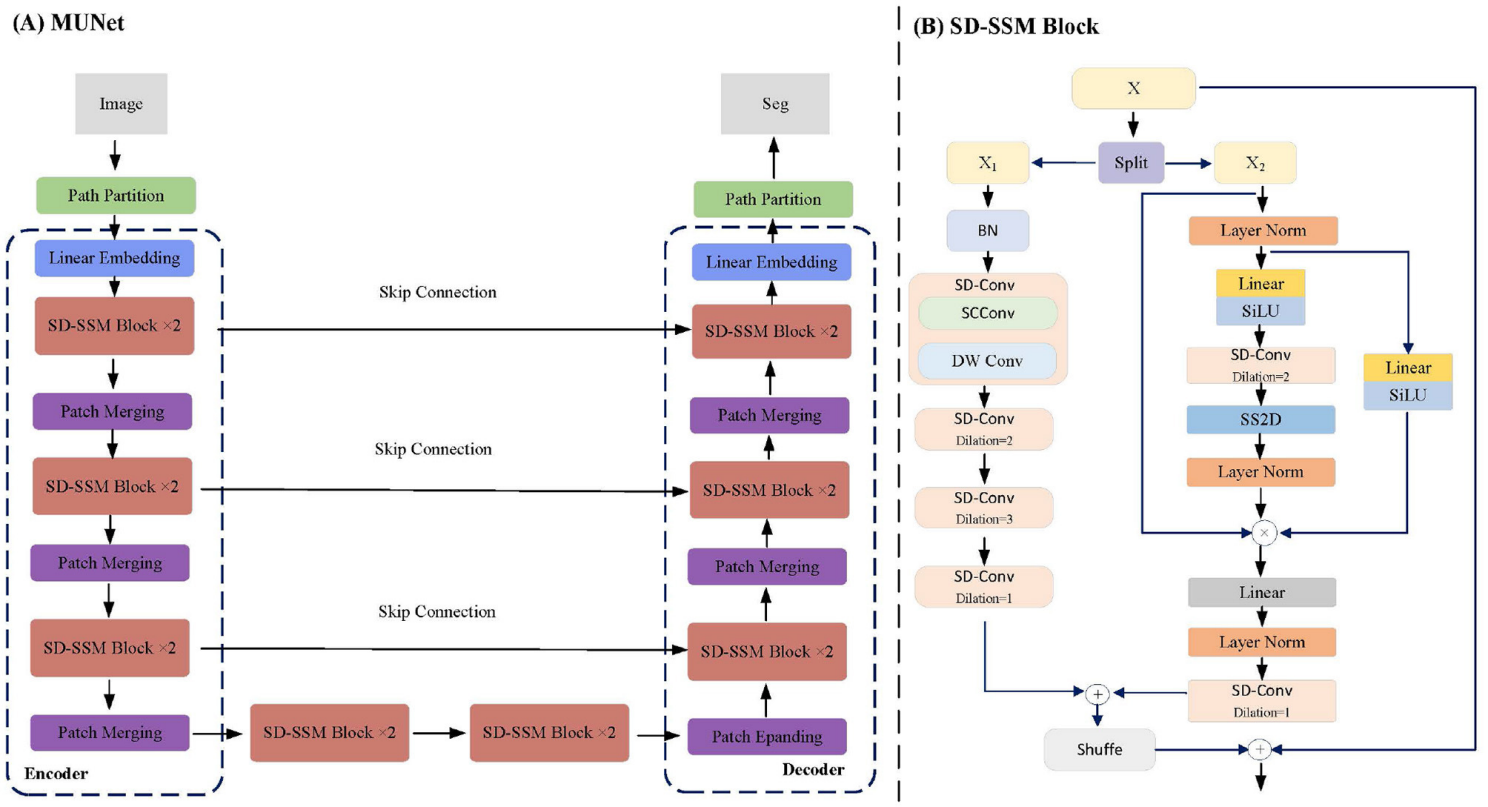
MUNet Architecture. **(A)** Illustrates the overall structure of MUNet, which follows an encoder-decoder design. The input image is processed through linear embedding, SD-SSM blocks, and multiple rounds of patch merging, with skip connections between the encoder and decoder layers facilitating information transfer. **(B)** Provides a detailed view of the internal structure of the SD-SSM block.

This paper proposes a network structure called MUNet, which combines SSM and the encoder-decoder architecture of UNet for efficient image segmentation. As shown in Figure 1A, MUNet first partitions the image into patches and performs linear embedding to obtain initial feature representations. These features are then processed by multiple SD-SSM block, where each module scans the feature sequences from different directions, capturing the global contextual information of the image for feature modeling and enhancement. The encoder gradually compresses the feature map to extract multi-scale information, while the Skip Connection passes features from the encoding process directly to the decoder to preserve image details. In the decoder, the network progressively restores the spatial resolution of the feature map and achieves accurate segmentation by integrating the multi-level features from the encoder. Patch Merging and Patch Expanding layers are used for feature compression and expansion, ensuring smooth information flow throughout the encoding-decoding process. Finally, through the combination of multiple SD-SSM block and skip connections, MUNet effectively captures both global and local features of the image, enhancing segmentation accuracy and efficiency.

### 3.3 SD-SSM block

The SD-SSM Block is a key module in the MUNet network, as shown in Figure 1B. Its structure comprises two main branches: *X*_1_ and *X*_2_. The *X*_1_ branch first applies batch normalization to the input features and then captures multi-scale information through multiple layers of dilated convolution using the SD-Conv module. The *X*_2_ branch normalizes the features through layer normalization and linear transformation, combined with the SiLU activation function to enhance non-linear representation capability. Subsequently, the features are processed through SD-Conv and SS2D modules to ensure the capture of global context information. The features from both branches are finally fused and connected to the residuals of the input features to enhance representational capacity.

**Figure 2:**
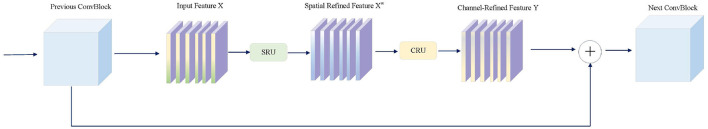
The architecture of SCConv integrated with the Spatial Reconstruction Unit (SRU) and the Channel Reconstruction Unit (CRU).

The core module of the SD-SSM Block, SD-Conv, is composed of SCConv (Spatial and Channel Reconstruction Convolution) (Li et al., 2023a) and DW Conv (Depthwise Separable Convolution) (Huang et al., 2023), as shown in Figure 2. SCConv reconstructs spatial and channel information through two units: the Spatial Reconstruction Unit (SRU), which suppresses spatial redundancy through a separated reconstruction method, and the Channel Reconstruction Unit (CRU), which eliminates channel redundancy through a split-transform-merge strategy. By integrating SCConv and DW Conv, SD-Conv effectively compresses spatial and channel redundancies among features, forming an efficient convolutional module. This module reduces redundant computations while preserving the representational capacity of the model, enabling better learning of key features in the image, particularly those in tumors, and enhancing the model's segmentation performance.

### 3.4 Skip connetions

Two SD-SSM Blocks are used in MUNet's encoder and decoder to effectively model both local and global features of the image. Each level of the encoder and decoder employs skip connections to mix multi-scale features with the upsampled output, enhancing spatial details by merging shallow and deep features. These skip connections ensure that high-resolution features from earlier layers of the encoder are preserved and fully utilized in the decoding process, maintaining crucial spatial information throughout the network and improving segmentation accuracy. This design enables MUNet to capture fine-grained details and contextual information simultaneously, achieving more precise and robust segmentation results, particularly in complex visual scenarios like tumor boundaries.

### 3.5 Loss function

In the domain of MRI brain tumor segmentation, the key evaluation metrics are the overlap between the segmentation results and the ground truth, as well as the accuracy and similarity of the boundaries. To address these metrics effectively, we design a weighted loss function that combines mIoU Loss, Dice Loss, and Boundary Loss. Each loss function optimizes a different aspect of the segmentation task, ensuring that the network achieves comprehensive and balanced performance.

The mean Intersection over Union (mIoU) loss is used to optimize the overlap between the predicted segmentation *P* and the ground truth *G*. It is defined as:


(12) $$\begin{array}{l} L_{\text{mIoU}}=1-\frac{1}{N}\overset{N}{\underset{i=1}{\sum }}\frac{\left|P_{i}\cap G_{i}\right|}{\left|P_{i}\cup G_{i}\right|} \end{array}$$

where *N* is the total number of pixels in the image, *P*_*i*_ is the set of pixels predicted as part of the tumor in the *i*-th class, and *G*_*i*_ is the set of ground truth pixels for the tumor in the *i*-th class. The mIoU loss penalizes regions where the segmentation and ground truth do not overlap well, focusing on improving the overall overlap accuracy.

The Dice loss aims to maximize the similarity between the predicted segmentation and the ground truth. It is defined as:


(13) $$\begin{array}{l} L_{\text{Dice}}=1-\frac{2\overset{N}{\underset{i=1}{\sum }}P_{i}G_{i}}{\overset{N}{\underset{i=1}{\sum }}P_{i}^{2}+\overset{N}{\underset{i=1}{\sum }}G_{i}^{2}} \end{array}$$

where *P*_*i*_ and *G*_*i*_ are the prediction and ground truth for each pixel. The Dice loss emphasizes the correct classification of tumor regions, focusing on the balance between false positives and false negatives, thus optimizing both sensitivity and precision.

The Boundary loss is used to refine the accuracy of the segmentation boundaries, which is crucial for brain tumor segmentation. It is defined as:


(14) $$\begin{array}{l} L_{\text{Boundary}}=\frac{\overset{N}{\underset{i=1}{\sum }}d_{\text{boundary}}\left(P_{i},G_{i}\right)}{N} \end{array}$$

where *d*_boundary_(*P*_*i*_, *G*_*i*_) is the distance between the predicted boundary and the ground truth boundary for pixel *i*, and *N* is the total number of pixels in the boundary region. The Boundary loss optimizes the fine details of the segmentation, ensuring that the predicted boundaries closely match the ground truth boundaries.

The final loss function is a weighted combination of the above three losses:


(15) $$\begin{array}{l} L_{\text{total}}=\alpha L_{\text{mIoU}}+\beta L_{\text{Dice}}+\gamma L_{\text{Boundary}} \end{array}$$

where α, β, γ are the weights that control the contribution of each loss term.

By combining these three loss components, the total loss function optimizes the segmentation task from multiple perspectives: enhancing overall overlap accuracy (mIoU), improving the similarity between predicted and actual tumor regions (Dice), and refining boundary precision (Boundary). This comprehensive approach allows for more accurate and effective segmentation results in MRI brain tumor analysis.

## 4 Experiments

### 4.1 Experimental setup

**Figure 3:**
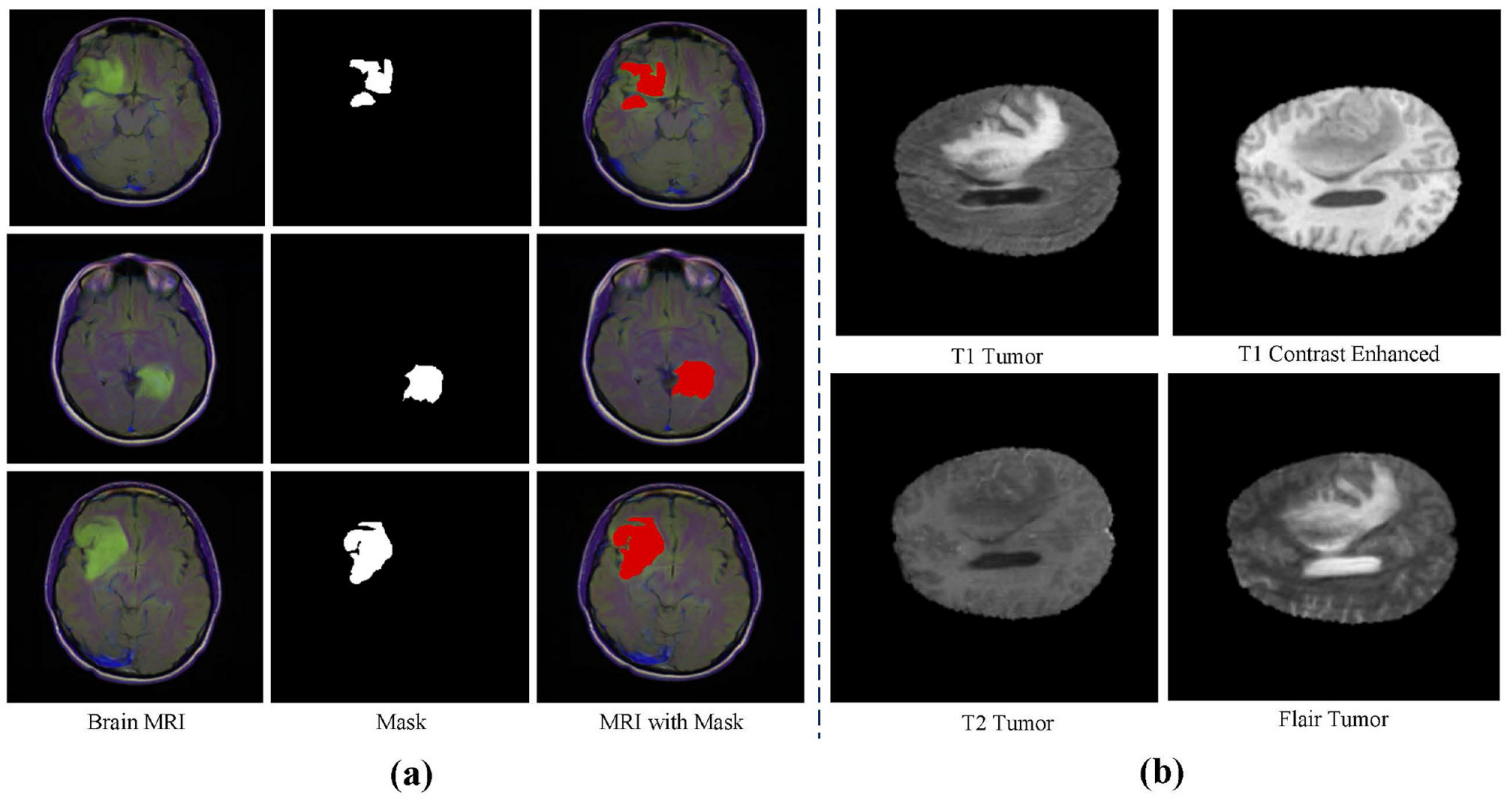
Dataset sample display. **(A)** LGG segmentation dataset display. **(B)** BraTS dataset display.

**Datasets** This study utilizes three main datasets for experiments: the BraTS2020 dataset, the BraTS2018 dataset (Bakas et al., 2017, 2018; Menze et al., 2014), and an independently validated LGG segmentation dataset (Buda et al., 2019). The LGG segmentation dataset is sourced from The Cancer Imaging Archive (TCIA) and includes MRI images from 110 patients in the Cancer Genome Atlas (TCGA) Low-Grade Glioma (LGG) collection, with a total of 3,929 images. These images are used for research on low-grade glioma segmentation, with the training set containing 2,750 images and the test set containing 1,179 images. Figure 3A shows an example from this dataset. The BraTS2020 dataset focuses on brain tumor segmentation, particularly for evaluating advanced methods in tumor segmentation using multimodal MRI scans. The BraTS2020 dataset provides a large training set of 369 MRI scan images and a validation set of 125 scans. Similarly, the BraTS2018 dataset is used for brain tumor segmentation, containing 285 training images and 66 validation images. Each MRI scan has a size of 240 × 240 × 155, with each case including multiple modalities such as T1, T1c, T2, and FLAIR. Figure 3B shows an example from the BraTS dataset.

**Experimental environment** The experiment was conducted on a high-performance server with the following hardware configuration: Intel Xeon Gold 6226R processor, NVIDIA Tesla V100 GPU (32GB memory), 128GB DDR4 RAM, and 1TB NVMe SSD storage, running Ubuntu 20.04 LTS as the operating system. For the software environment, PyTorch 1.10 was used as the deep learning framework, with CUDA 11.4 and cuDNN 8.2 for acceleration, and Python 3.8 as the programming language. The scientific computing libraries included NumPy 1.21 and SciPy 1.7, while Pandas 1.3 and Matplotlib 3.4 were used for data processing and visualization. Additionally, OpenCV 4.5 was installed for image processing, and scikit-learn 0.24 for data analysis and model evaluation. All software packages were managed using the conda environment management tool to ensure reproducibility and compatibility of dependencies.

**Evaluation metrics** The evaluation metrics used in this paper include Kappa (k), Dice Similarity Coefficient (DSC), Intersection over Union (IoU), Sensitivity (S), Precision (P), Specificity (Sp), Accuracy (A), and Balanced Accuracy (BA).

Kappa is used to measure the agreement between the predicted and actual classifications, considering the possibility of the agreement occurring by chance.


(16) $$\begin{array}{l} k=\frac{P_{o}-P_{e}}{1-P_{e}} \end{array}$$

Where: *P*_*o*_ is the observed agreement (the proportion of correct predictions). *P*_*e*_ is the expected agreement (the proportion of correct predictions by chance).

DSC is used to measure the degree of overlap between two sets, while IoU measures the ratio of the intersection and the union between the predicted and the ground truth regions.


(17) $$\begin{array}{l} DSC=\frac{2\times |X\cap Y|}{\left|X|+|Y\right|} \end{array}$$

(18) $$\begin{array}{l} IoU=\frac{\left|X\cap Y\right|}{\left|X\cup Y\right|} \end{array}$$

where *X* and *Y* represent the sets of predicted and ground truth pixels, respectively.

Sensitivity (S) measures the model's ability to correctly identify positive instances, precision (P) represents the correctness of the predicted positive instances, specificity (Sp) measures the ability to correctly identify negative instances, and accuracy (A) measures the overall correctness of the model's predictions.


(19) $$\begin{array}{l} S=\frac{TP}{TP+FN} \end{array}$$

(20) $$\begin{array}{l} P=\frac{TP}{TP+FP} \end{array}$$

(21) $$\begin{array}{l} Sp=\frac{TN}{TN+FP} \end{array}$$

(22) $$\begin{array}{l} A=\frac{TP+TN}{TP+TN+FP+FN} \end{array}$$

where *TP* stands for true positives, *TN* stands for true negatives, *FP* stands for false positives, and *FN* stands for false negatives.

Balanced Accuracy is used to account for imbalanced data, providing the average of sensitivity and specificity.


(23) $$\begin{array}{l} BA=\frac{S+Sp}{2} \end{array}$$

Hausdorff95 is a metric commonly used in segmentation tasks to measure the spatial distance between the predicted boundary and the ground truth boundary. Unlike the traditional Hausdorff Distance, which considers the maximum distance between two point sets, Hausdorff95 focuses on the 95th percentile distance, effectively reducing the influence of outliers and providing a more robust evaluation for medical image segmentation tasks where extreme outliers might distort the overall assessment.


(24) $$\begin{array}{l} H_{95}\left(A,B\right)=\max\left\{h_{95}\left(A,B\right),h_{95}\left(B,A\right)\right\} \end{array}$$

where, *h*_95_(*A, B*) represents the 95th percentile of the set of minimum distances from points in set *A* to the closest points in set *B*. *A* and *B* are typically the sets of points that represent the boundaries of the ground truth segmentation and the predicted segmentation, respectively. This measure ensures that 95% of the points on the predicted boundary are within a certain distance from the ground truth boundary, making it a more resilient metric for segmentation accuracy, particularly in scenarios where small boundary discrepancies are permissible.

### 4.2 Results

In this paper, the MUNet model demonstrated outstanding performance on both the BraTS2020 and BraTS2018 datasets, significantly surpassing other existing models. As shown in Table 1, MUNet's performance on the three key metrics-enhancing tumor (ET), whole tumor (WT), and tumor core (TC)-is highlighted.

**Table 1:** Comparison of different methods using DSC and Hausdorff95 metrics for BraTS2020 dataset and BraTS2018 dataset.

**Method**	**DSC (BraTS2020)**			**Hausdorff95 (BraTS2020)**			**DSC (BraTS2018)**			**Hausdorff95 (BraTS2018)**		
	**ET**	**WT**	**TC**	**ET**	**WT**	**TC**	**ET**	**WT**	**TC**	**ET**	**WT**	**TC**
**U-Net**	0.783	0.882	0.801	5.835	5.447	7.123	0.724	0.851	0.747	6.554	12.645	11.035
**Attention U-Net** (Oktay et al., 2018)	0.775	0.859	0.798	4.586	5.253	7.174	0.765	0.869	0.758	7.596	10.253	11.174
**ResU-Net** (Maji et al., 2022)	0.812	0.895	0.813	4.759	5.789	6.597	0.795	0.892	0.758	6.993	8.597	10.046
**FE-HU-NET** (Nizamani et al., 2023)	0.802	0.870	0.815	4.859	5.253	5.764	0.742	0.872	0.745	5.894	8.243	9.765
**HC-Mamba** (Xu, 2024)	0.795	0.899	0.812	5.358	4.125	7.766	0.812	0.883	0.787	5.459	6.248	9.764
**Mamba-UNet** (Wang et al., 2024)	0.813	0.902	0.798	3.389	4.243	6.766	0.802	0.906	0.801	5.127	6.873	6.766
**SwimUNet** (Cao et al., 2022)	0.792	0.892	0.745	5.347	6.729	8.588	0.776	0.847	0.759	6.798	8.257	8.712
**TransUNet** (Chen et al., 2021)	0.798	0.877	0.712	3.598	5.871	6.766	0.762	0.850	0.765	6.389	8.247	8.153
**MUNet**	**0.835**	**0.915**	**0.823**	**2.421**	**3.755**	6.437	**0.815**	**0.901**	**0.815**	**4.389**	**6.243**	**6.152**

The optimal results are indicated in bold.

On the BraTS2020 dataset, MUNet exhibited a notable improvement over other methods. Specifically, for ET segmentation, MUNet achieved a DSC score of 0.835, approximately 6.6% higher than the traditional U-Net. For WT segmentation, MUNet reached a DSC score of 0.915, outperforming ResU-Net by about 2.2%. Additionally, MUNet showed clear advantages in TC segmentation, improving by around 2.7% compared to U-Net. In terms of boundary accuracy, MUNet also performed exceptionally well, with the Hausdorff95 distance for ET reduced by nearly 58% compared to U-Net, indicating significant improvements in boundary capturing and morphological recognition.

On the BraTS2018 dataset, MUNet also exhibited substantial improvements. For ET segmentation, MUNet's DSC score increased by about 12.5% compared to traditional U-Net. WT segmentation accuracy improved by around 5.9%, meaning MUNet can more effectively capture the global morphological characteristics of tumors. Notably, MUNet also showed significant improvements in Hausdorff95 distance, reducing the ET score by approximately 37% compared to other models.

This result also indicates that, compared to existing CNN-based and Transformer-based models, MUNet demonstrates superior performance in tumor segmentation tasks. For example, although ResU-Net improves feature propagation through residual connections, it still falls short when handling complex MRI images. MUNet, by integrating both global and local features and leveraging the SD-SSM module to effectively capture detailed information, significantly improves segmentation accuracy. In comparison to SwimUNet, MUNet also shows a clear advantage in detail processing. While SwimUNet enhances global context information, it does not perform as well as MUNet in the reconstruction of fine boundaries. MUNet ensures precise boundary modeling through skip connections and multi-layer SD-Conv modules, resulting in a lower Hausdorff95 distance and higher segmentation accuracy.

**Figure 4:**
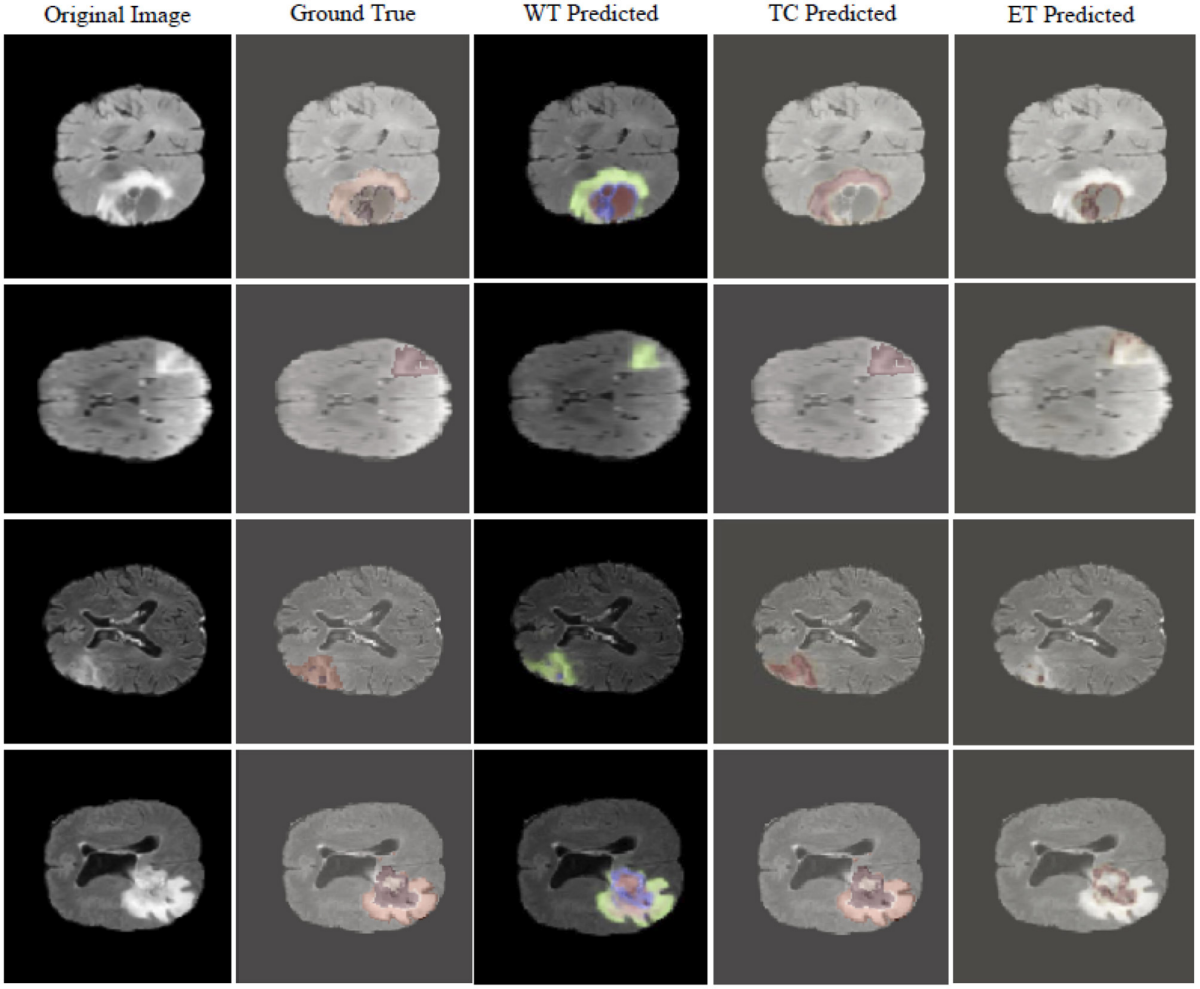
Display of MUNet segmentation performance on the BraTS dataset.

Figure 4 visualizes the tumor detection results of MUNet for brain tumors. As seen in the figure, MUNet demonstrates remarkable accuracy in segmenting tumor regions, particularly excelling in delineating boundary areas, where it clearly outperforms traditional models. The segmentation results not only provide a clear depiction of the tumor's morphological features but also retain critical detailed information. Additionally, MUNet exhibits excellent global consistency in segmenting both WT and TC, with the tumor contours and core regions represented comprehensively and accurately. These results highlight MUNet's strong capability in handling complex brain tumor shapes and its potential for precise tumor analysis in medical imaging.

**Computational complexity analysis** As shown in Table 1, we further compared the computational complexity of MUNet with other existing models, specifically considering the number of floating-point operations (FLOPs) and the number of model parameters. The data in the table clearly show that MUNet has a significant advantage in both FLOPs and parameter count. Firstly, MUNet has 140.97 GFLOPs, which is notably lower than most traditional models, especially SwimUNet and TransUNet, which have 370.31 GFLOPs and 390.76 GFLOPs, respectively. This indicates that MUNet is more computationally efficient and can perform the same tasks with fewer computational resources, reducing both computational cost and runtime. Additionally, MUNet has 7.27M parameters, which is significantly fewer compared to models such as ResU-Net (25.75M), SwimUNet (25.18M), and TransUNet (20.45M). The smaller parameter count not only helps accelerate model training but also reduces memory usage, facilitating efficient deployment even under hardware constraints.

### 4.3 Ablation study

In the ablation experiments of this paper, we conducted a detailed analysis of MUNet's performance on the BraTS2020 and BraTS2018 datasets by progressively adding each of its modules. Additionally, we analyzed the impact of the proposed loss functions on MUNet's performance.

#### 4.3.1 Ablation experiments between components

**Performance on the BraTS2020 dataset** As shown in Table 1, the baseline U-Net model achieved DSC scores of 0.783, 0.882, and 0.801 for ET, WT, and TC segmentation, respectively, with Hausdorff95 distances of 5.835, 5.447, and 7.123. With the introduction of the SD-SSM Block, the model's ability to capture global and local features improved, resulting in increased DSC scores of 0.795, 0.895, and 0.805 for ET, WT, and TC, along with a reduction in Hausdorff95 distances. Notably, the Hausdorff95 distance for WT decreased from 5.447 to 4.358, indicating a significant improvement in boundary accuracy. When the SD-Conv structure was further added, the model's performance improved again, with DSC scores increasing to 0.815, 0.899, and 0.810 for ET, WT, and TC, respectively, and a reduction in Hausdorff95 distances across all metrics, especially for ET, which decreased to 4.524. This improvement suggests that the SD-Conv structure helped reduce feature redundancy and enhance computational efficiency, contributing to better segmentation accuracy while maintaining high-resolution features. Finally, after the introduction of the newly designed loss function, the model reached optimal performance. The DSC scores for ET, WT, and TC rose to 0.835, 0.915, and 0.823, respectively, significantly outperforming all previous combinations. Notably, the Hausdorff95 distance for ET decreased to 2.421, a reduction of approximately 58%. This demonstrates that the new loss function played a crucial role in optimizing the overlap, similarity, and boundary accuracy of the segmentation results, significantly improving the model's ability to handle complex tumor morphologies. From these ablation experiment results, it can be seen that the SD-SSM Block and SD-Conv modules work synergistically, not only improving the model's ability to capture global and local features but also enhancing efficiency by reducing feature redundancy. Moreover, the new loss function played a critical role in improving boundary accuracy and preserving fine details. Together, these components have enabled MUNet to demonstrate significant advantages in brain tumor segmentation tasks.

**Performance on the BraTS2018 dataset** In the ablation experiments on the BraTS2018 dataset, the results followed a similar trend to those observed in the BraTS2020 dataset. As shown in Table 2, the baseline U-Net model achieved DSC scores of 0.724, 0.851, and 0.747 for ET, WT, and TC segmentation, respectively, with corresponding Hausdorff95 distances of 6.554, 12.645, and 11.035, serving as the baseline performance. When the SD-SSM Block was added, although the DSC for WT slightly decreased to 0.779, the DSC for ET and TC improved slightly to 0.764 and 0.728, respectively. In addition, there was an improvement in Hausdorff95 distances, particularly for WT, where the distance decreased from 12.645 to 11.273, indicating some enhancement in boundary accuracy. With the introduction of the SD-Conv module, the overall performance of the model improved significantly. The DSC scores for ET, WT, and TC increased to 0.796, 0.885, and 0.787, respectively, and the Hausdorff95 distances dropped considerably, especially for WT, where it decreased from 11.273 to 8.597. This suggests that the SD-Conv module greatly contributed to feature extraction and boundary handling. Finally, when the new loss function was introduced, the model reached its optimal performance. The DSC scores for ET, WT, and TC rose to 0.815, 0.901, and 0.815, respectively, and the Hausdorff95 distances significantly decreased across all metrics, particularly for ET, where the distance dropped to 4.389. This indicates that the new loss function greatly enhanced the model's segmentation accuracy, especially in handling boundaries and complex tumor morphologies.

**Table 2:** Comparison and analysis of dataset model efficiency.

**Methods**	**FLOPs (GFLOPs)**	**Number of parameters (Millions)**
U-Net	142.05	6.53M
Attention U-Net	156.21	7.49M
ResU-Net	242.96	25.75M
FE-HU-NET	241.97	11.75M
HC-Mamba	169.25	9.38M
Mamba-UNet	199.89	22.65M
SwimUNet	370.31	25.18M
TransUNet	390.76	20.45M
MUNet	**140.97**	7.27M

The optimal results are indicated in bold.

#### 4.3.2 Ablation experiments on the loss function

**Performance on the BraTS2020 dataset** As shown in Table 5, the complete MUNet model achieved a DSC score of 0.835 for ET segmentation. However, when the mIoU loss function was removed, the DSC significantly dropped by around 20%, down to 0.665. At the same time, the Hausdorff95 distance increased substantially from 2.421 to 6.586, almost a 2.7-fold increase, indicating the critical role of mIoU in enhancing overall segmentation accuracy. When the Dice loss was removed, the DSC for ET decreased to 0.709, about 15% lower than the complete model, and the Hausdorff95 distance increased to 8.687, highlighting the importance of Dice loss in improving regional similarity. Similarly, removing the Boundary loss resulted in a DSC drop to 0.642, a reduction of approximately 23%, while the Hausdorff95 distance increased to 9.126, nearly quadrupling, emphasizing the significance of Boundary loss in ensuring segmentation boundary accuracy. For WT and TC segmentation, similar trends were observed. Removing the mIoU loss led to a roughly 25% decrease in WT's DSC, and the Hausdorff95 distance more than doubled. Removing Dice and Boundary losses resulted in approximately 20% reductions in segmentation accuracy, with significant increases in Hausdorff95 distance, indicating that each loss function plays a key role in optimizing performance for different segmentation targets.

**Table 3:** Ablation experiments on different combinations of MUNet on the BraTS2020 dataset.

**Method**	**DSC**			**Hausdorff95**		
	**ET**	**WT**	**TC**	**ET**	**WT**	**TC**
**U-Net**	0.783	0.882	0.801	5.835	5.447	7.123
**U-Net + SD-SSM Block**	0.795	0.895	0.805	5.105	4.358	6.578
**U-Net + SD-SSM Block + SD-Conv**	0.815	0.899	0.810	4.524	3.698	6.581
**U-Net + SD-SSM Block + SD-Conv + Loss function**	**0.835**	**0.915**	**0.823**	**2.421**	**3.755**	**6.437**

The optimal results are indicated in bold.

**Table 4:** Ablation experiments on different combinations of MUNet on the BraTS2018 dataset.

**Method**	**DSC**			**Hausdorff95**		
	**ET**	**WT**	**TC**	**ET**	**WT**	**TC**
**U-Net**	0.724	0.851	0.747	6.554	12.645	11.035
**U-Net + SD-SSM Block**	0.764	0.779	0.728	6.586	11.273	10.175
**U-Net + SD-SSM Block + SD-Conv**	0.796	0.885	0.787	5.993	8.597	7.046
**U-Net + SD-SSM Block + SD-Conv + Loss function**	**0.815**	**0.901**	**0.815**	**4.389**	**6.243**	**6.152**

The optimal results are indicated in bold.

**Table 5:** Ablation experiments on the loss function of MUNet.

**Method**	**DSC (BraTS2020)**			**Hausdorff95 (BraTS2020)**			**DSC (BraTS2018)**			**Hausdorff95 (BraTS2018)**		
	**ET**	**WT**	**TC**	**ET**	**WT**	**TC**	**ET**	**WT**	**TC**	**ET**	**WT**	**TC**
**MUNet**	**0.835**	**0.915**	**0.823**	**2.421**	**3.755**	**6.437**	**0.815**	**0.901**	**0.815**	**4.389**	**6.243**	**6.152**
**w/o** ${ℒ}_{\text{mIoU}}$	0.665	0.689	0.728	6.586	11.253	15.174	0.710	0.724	0.763	7.258	15.254	14.184
**w/o** ${ℒ}_{\text{Dice}}$	0.709	0.714	0.708	8.687	10.543	11.258	0.684	0.692	0.719	8.573	12.574	13.245
**w/o** ${ℒ}_{\text{Boundary}}$	0.642	0.671	0.665	9.126	10.374	12.149	0.601	0.679	0.712	10.589	14.153	15.766

The optimal results are indicated in bold.

**Performance on the BraTS2018 dataset** For the BraTS2018 dataset, the complete MUNet model achieved a DSC score of 0.815 for ET segmentation. After removing the mIoU loss, the DSC dropped by approximately 13%, to 0.710, while the Hausdorff95 distance increased by 65%, from 4.389 to 7.258. This shows that mIoU is equally crucial for improving global segmentation performance on this dataset. Removing the Dice loss reduced the DSC for ET to 0.684, a decrease of around 16%, and the Hausdorff95 distance increased to 8.573, nearly doubling. Similarly, removing the Boundary loss led to a 26% reduction in ET's DSC, while the Hausdorff95 distance increased to 10.589, almost 2.4 times higher, demonstrating the essential role of Boundary loss in capturing accurate boundaries in complex tumor morphologies. The segmentation for WT and TC also showed similar declines. Removing the mIoU loss resulted in a 20% reduction in WT's DSC, while the Hausdorff95 distance roughly doubled. Removing Dice and Boundary losses decreased segmentation accuracy by about 20%, with significant increases in Hausdorff95 distance, confirming the multi-dimensional improvement in segmentation performance provided by these loss functions.

### 4.4 Independent validation

**Figure 5:**
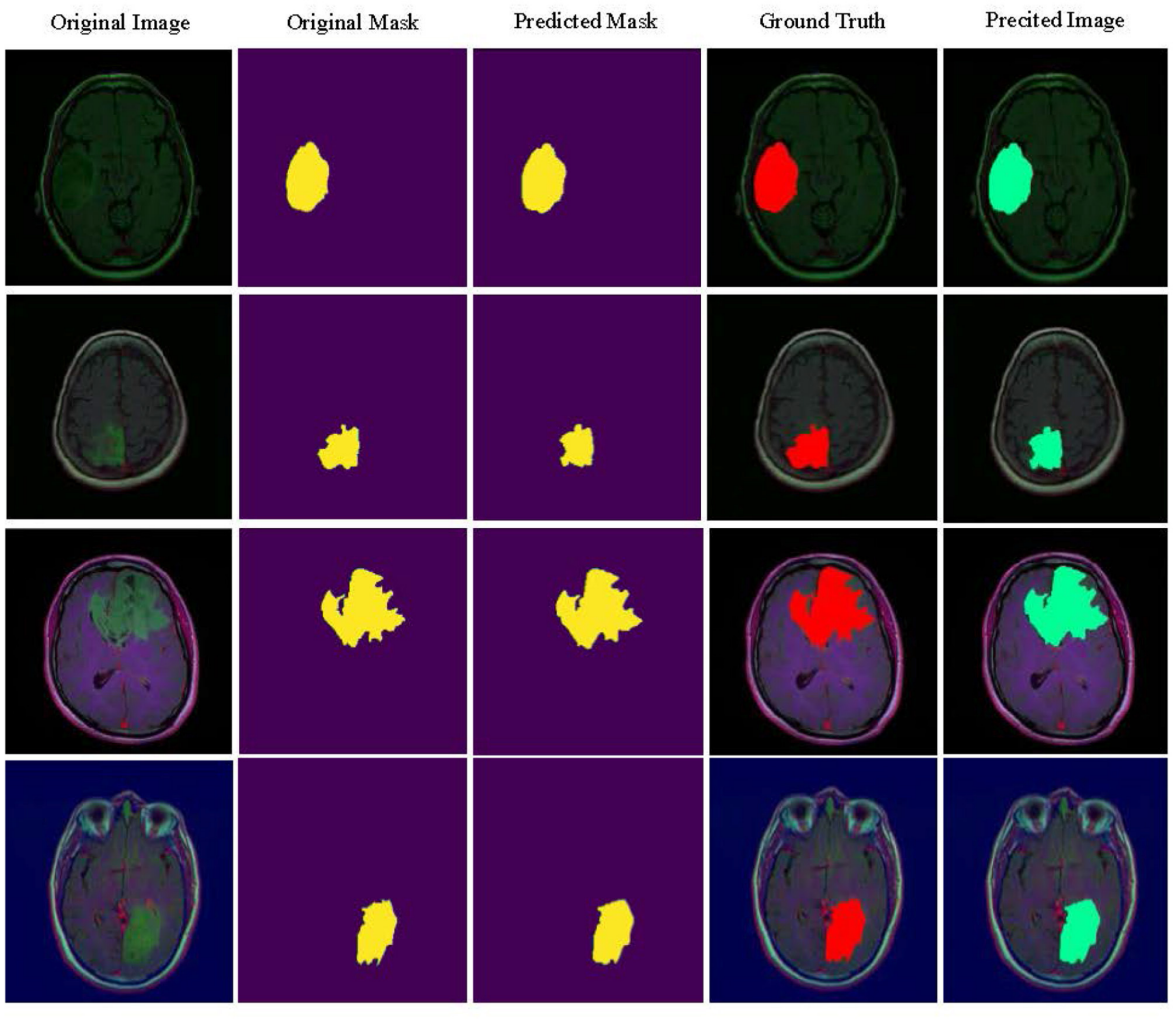
Display of MUNet segmentation performance on the LGG segmentation dataset.

The independent validation on the LGG segmentation dataset, as shown in Table 6, demonstrates that the MUNet model performs well across several key performance metrics. The model exhibits high accuracy and balanced accuracy, along with excellent performance in specificity and AUC, indicating its robustness and generalization ability in the LGG segmentation task. These results validate the effectiveness of MUNet in handling medical image segmentation tasks. Figure 5 visualizes the LGG segmentation dataset, and from the figure, the segmentation results of the model can be intuitively observed. MUNet is able to accurately identify brain tumor segmentation regions, and the overlap between the segmentation results and the ground truth is highly consistent, further demonstrating the model's superior performance and reliability. This high-quality segmentation result also lays a solid foundation for automated processing in medical image analysis.

**Table 6:** MUNet independent validation on the LGG segmentation dataset.

**Method**	**Kappa**	**DSC**	**IOU**	**Sensitivity**	**Specificity**	**Precision**	**Accuracy**	**BA**	**AUC**
**MUNet**	0.678	0.702	0.581	0.658	0.975	0.762	0.981	0.808	0.812

### 4.5 Limitations and future work

Although MUNet performs well in the task of brain tumor segmentation, there are still some limitations that require further research and improvement. Firstly, while the SD-SSM block in MUNet effectively combines global and local features, the model's accuracy may decline when dealing with extremely complex or highly heterogeneous tumor regions. In such cases, unclear tumor boundaries or irregular shapes may result in insufficient precision in segmentation. Future work could focus on enhancing boundary detection mechanisms to improve boundary recognition in complex tumor regions.

Secondly, as a black-box model, MUNet lacks interpretability, which may limit its widespread application in clinical settings. Although MUNet performs excellently in brain tumor segmentation tasks, in clinical practice, doctors often need to understand the decision-making process of the model, especially when faced with critical diagnoses (Dasanayaka et al., 2022a). Future research could focus on designing transparent reasoning processes or explanation modules within the model, allowing doctors to clearly understand each decision step and the weight distribution, thereby increasing trust in the model's predictions.

In addition, although MUNet has achieved good performance on the current dataset, it still faces the issue of overfitting, especially when the data is limited or the data distribution is imbalanced. Overfitting may result in good performance on the training set, but poor predictive performance on unseen data in practical applications. To reduce the risk of overfitting, future research can enhance the diversity of the dataset by increasing the amount of annotated data or employing data augmentation techniques to expand the training set. Meanwhile, regularization methods can help reduce model complexity and decrease the probability of overfitting. Additionally, transfer learning could be employed, where the model is pre-trained on large publicly available datasets and then fine-tuned for the specific brain tumor segmentation task, thus improving the model's generalization ability.

## 5 Conclusion

This paper presents a novel network framework named MUNet, which combines the advantages of UNet and Mamba to achieve efficient and accurate brain tumor segmentation. By introducing the SD-SSM module, which utilizes selective scanning and state space modeling, MUNet can capture both global and local features of images, thereby improving segmentation accuracy. Additionally, the integration of the SD-Conv structure reduces feature redundancy without increasing the number of parameters, enhancing the overall efficiency of the model. Experimental results show that MUNet outperforms existing methods on the BraTS2020 and BraTS2018 datasets, achieving superior segmentation accuracy. Moreover, MUNet demonstrates excellent generalization capabilities when validated on the independent LGG segmentation dataset, further proving its effectiveness in various medical imaging scenarios. Future work may focus on extending the application of MUNet to other imaging modalities and exploring more advanced learning strategies to further enhance its clinical applicability.

## Data Availability

Publicly available datasets were analyzed in this study. This data can be found here: BraTS2020 Dataset: https://www.kaggle.com/datasets/awsaf49/brats20-dataset-training-validation.BraTS2018, Dataset: https://www.kaggle.com/datasets/anassbenfares/brats2018.LGGSegmentation, and Dataset: https://www.kaggle.com/datasets/mateuszbuda/lgg-mri-segmentation.
